# Low public awareness of the human papillomavirus (HPV) vaccine and associated challenges in Pakistan: an editorial

**DOI:** 10.1097/MS9.0000000000004578

**Published:** 2025-12-18

**Authors:** Muhammad Furqan, Sadia Binte Rahim, Muhammad U. Haider

**Affiliations:** a Department of Medicine, King Edward Medical University, Nelagumbad, Anarkali, Lahore, Pakistan; b Department of Medicine, Mymensingh Medical College Hospital, Bangladesh; c Department of Internal Medicine, Geisinger Health System, Wilkes-Barre, Pennsylvania

**Keywords:** challenges, HPV vaccine, human papillomavirus, Pakistan, public awareness

## Abstract

Cervical cancer is the third leading cause of cancer-related deaths among women in Pakistan, with over 5000 new cases and 3200 deaths annually. In September 2025, the Federal Directorate of Immunization, supported by World Health Organization, Gavi, and UNICEF, launched Pakistan’s first human papillomavirus (HPV) vaccination campaign targeting 13 million girls aged 9–14 years. Despite the proven efficacy of a single vaccine dose, awareness remains critically low, with studies showing that most women have little knowledge of HPV or its vaccine. Cultural barriers, misinformation, limited healthcare infrastructure, and inadequate education pose significant challenges, highlighting the need for sustained awareness and community engagement.

Cervical cancer is a significant public health concern in Pakistan, where it ranks as the third leading cause of cancer-related deaths among Pakistani women^[^1^]^. The age-standardized incidence rate ranges from 5.20 to 8.54 per 100 000 women^[^2^]^, with over 5000 new cases and around 3200 deaths reported annually^[^1^]^. The epidemiological trends of cervical cancer in Pakistan demonstrate significant regional and age-related variation. Evidence from the Karachi Cancer Registry showed an increase in the age-standardized incidence rate (ASIR) in Karachi South by approximately 10% between 1995 and 1997 (6.81 per 100 000) and 1998–2002 (7.5 per 100 000)^[^3^]^. Over this same period, the mean age at diagnosis dropped slightly from 53 to 51 years, with most cases presenting at an advanced stage^[^3^]^. Furthermore, a systematic review combining data from Karachi, Punjab, and other centers estimated the adjusted national ASIR falls between 5.2 and 8.4 per 100 000, suggesting considerable geographic heterogeneity and calculating about 6166 new cases annually. These figures underscore both the rising incidence observed over time in some regions and the disease’s disproportionate impact on middle-aged women^[^2^]^. The high mortality burden is attributed to limited access to regular screening and human papillomavirus (HPV) vaccination, coupled with poor knowledge and awareness about cervical cancer risks, symptoms, and the importance of early detection^[^4^]^. Persistent infection with high-risk HPV types is the primary cause of this disease. In response, Pakistan’s Federal Directorate of Immunization (FDI), with support from the World Health Organization (WHO), Gavi, and United Nations Children’s Fund (UNICEF), has launched its first vaccination campaign to protect 13 million girls aged 9–14 years from cervical cancer^[^5^]^.

Although a single dose of the HPV vaccine can prevent most cervical cancers, a critical obstacle to the campaign’s success is low public awareness and acceptance of HPV vaccination. In a Karachi hospital study, only about 20.6% of women knew HPV was a cervical cancer risk factor, and merely 25.5% knew a preventive vaccine existed^[^6^]^. In a cross-sectional study in Punjab, it was reported that about 77% of the participants had never heard of HPV infection, and about 90% were unaware of the vaccine. The study also noted that urban and educated women were more aware of cervical cancer prevention and were more than willing to get vaccinated^[^7^]^. The causes of this low awareness are multifaceted. Pakistan has never had a national HPV immunization program until now, so information about the vaccine has been sparse. Only relatively recently have doctors and the media begun discussing it after the government’s announcement. Even among healthcare providers, there have been gaps, as many clinicians historically received little training about HPV vaccination, and have not routinely counseled patients on it. Until the campaign, the only HPV vaccines available in Pakistan were via private distributors at high cost, effectively excluding most families. Rural areas have abysmal access to primary care services^[^8^]^. Cultural and religious factors, as well as misinformation, exacerbate these challenges. In Pakistan, there are persistent myths (for instance, that the vaccine causes infertility or is only for sexually active girls) which sow doubt about HPV vaccination leading to “vaccination hesitancy”^[^6^]^. All these factors, along with a poor literacy rate, are significant challenges that compromise the success of the HPV vaccination program.

To bridge this awareness gap, public health experts have proposed a range of supportive measures, such as nationwide awareness campaigns by mobilizing mass media and local influencers with television (TV), radio, and social media messages that clearly state that HPV vaccination is safe and cancer-preventing^[^9^]^. Public education drives in Urdu and regional languages, including mobile phone alerts and TV spots, can explain what HPV is and why vaccination matters. Schools and community centers can hold question-and-answer sessions that allow parents to raise concerns directly with health professionals. Community engagement events involving health workers and cervical cancer survivors can further motivate families^[^10^]^. In addition, endorsements from religious authorities, including scholarly fatwas confirming that the vaccine is permissible, would help dispel misconceptions and build credibility. The effective implementation of cervical cancer prevention efforts requires a strategic division into short-term and long-term actions with clearly designated implementers to improve both clarity and feasibility. In the short term, the priority is rapid, widespread communication led by the Ministry of National Health Services, Regulations and Coordination, the FDI, and partners such as WHO and UNICEF. These efforts must include intensified social media campaigns, urgent TV/radio messaging coordinated by the Pakistan Electronic Media Regulatory Authority, and community-level myth-busting led by Lady Health Workers and non-governmental organizations (NGOs). Schools should host parent question and answer forums, and the Council of Islamic Ideology must secure public endorsement for HPV vaccination from religious scholars. In the long term, sustainable structural changes are needed, such as incorporating HPV and cervical cancer prevention education into school curricula through the Ministry of Education, integrating HPV counseling into routine primary-care visits via provincial health departments, and formally embedding HPV vaccination into Pakistan’s Expanded Programme on Immunization. Continued partnerships with NGOs and community organizations will also be essential for maintaining awareness and expanding access to screening and vaccination services. These tiered, implementer-specific strategies clarify not only who should act but also how these efforts can be coordinated and sustained over time.

Beyond the immediate operational hurdles, Pakistan’s HPV vaccine introduction highlights a persistent global health challenge known as the translational gap, which is the difficulty of turning major scientific advances into effective population-level benefits. This gap is evident in examples such as the RTS,S malaria vaccine, whose real-world impact is limited by multidose schedules, fragile cold chain systems, and parental concerns, as well as point-of-care diagnostics for HIV and tuberculosis that struggle due to weak integration into clinics, limited training, and supply chain barriers^[^11^]^. These cases show that scientific breakthroughs alone cannot guarantee public health gains without trusted and functional delivery systems. The rapid progress of computational tools such as AlphaFold, which has significantly advanced early vaccine and drug discovery, demonstrates how quickly upstream innovation is moving, yet the full value of these advances depends on effective downstream implementation, clear communication, and community acceptance^[^12^]^. Addressing this last-mile challenge requires a strong emphasis on implementation science. The Consolidated Framework for Implementation Research offers a structured way to identify the many factors that influence uptake, including the nature of the intervention, the wider cultural and policy environment, and the readiness of health facilities^[^13^]^. In parallel, the RE-AIM (Reach, Effectiveness, Adoption, Implementation, and Maintenance) model provides a practical approach for evaluating reach, effectiveness, adoption, implementation, and long-term maintenance, ensuring that successful interventions extend beyond controlled research settings and achieve sustained impact across diverse populations^[^14^]^. Together, these frameworks help bridge the gap between scientific discovery and real-world public health benefit. Successful examples from other low and middle-income countries, including Rwanda’s high-coverage school-based HPV vaccination program and the scale-up of antiretroviral therapy in sub-Saharan Africa driven by political commitment and community-based support, demonstrate how system readiness determines impact^[^15^]^. These steps, taken before and during the campaign, can build trust and ensure Pakistan’s daughters benefit fully from this historic initiative. However, many measures were not systematically implemented before the launch, contributing to confusion, misinformation, and anxiety. Earlier awareness efforts could have reduced these concerns, and now, integrating clear communication and community engagement throughout the campaign is essential to rebuilding trust. Future research must therefore move beyond proving efficacy to optimizing implementation, focusing on context-specific communication strategies, stronger health systems, and interdisciplinary work that connects upstream scientific innovation with practical, community-centered delivery models to advance equitable global health outcomes.

## Data Availability

The datasets are available from the corresponding author on reasonable request.

## Research registration unique identifying number (UIN)

This is not an original research project involving human participants in an interventional or an observational study but a case report. This registration was not required.
